# Draft Genome Sequence of Weissella cibaria P71, a Promising Aquaculture Probiotic Strain Isolated from Common Octopus (Octopus vulgaris)

**DOI:** 10.1128/MRA.00792-21

**Published:** 2021-12-09

**Authors:** Diogo Contente, Javier Feito, Lara Díaz-Formoso, Beatriz Gómez-Sala, Juan Borrero, Nuria Peña, Gilberto Igrejas, Patrícia Poeta, Pablo E. Hernández, Estefanía Muñoz-Atienza, Luis M. Cintas

**Affiliations:** a Grupo de Seguridad y Calidad de los Alimentos por Bacterias Lácticas, Bacteriocinas y Probióticos, Sección Departamental de Nutrición y Ciencia de los Alimentos, Facultad de Veterinaria, Universidad Complutense de Madrid, Madrid, Spain; b Microbiology and Antibiotic Resistance Team, Department of Veterinary Sciences, University of Trás-os-Montes and Alto Douro, Vila Real, Portugal; c APC Microbiome Ireland, University College Cork, Cork, Ireland; d Teagasc Food Research Centre, Moorepark, Fermoy, Cork, Ireland; e Functional Genomics and Proteomics Unit, University of Trás-os-Montes and Alto Douro, Vila Real, Portugal; f Department of Genetics and Biotechnology, University of Trás-os-Montes and Alto Douro, Vila Real, Portugal; g Associated Laboratory for Green Chemistry, University NOVA of Lisbon, Caparica, Portugal; University of Southern California

## Abstract

Weissella cibaria P71 is a lactic acid bacterium that was isolated from common octopus (Octopus vulgaris) and previously showed interesting probiotic properties for turbot (Scophthalmus maximus L.) farming. The draft genome sequence of this strain provides further data to support its potential as a probiotic for aquaculture.

## ANNOUNCEMENT

Weissella cibaria, which was described in 2002 (1), has attracted great interest due to its antimicrobial, technological, nutritional, and probiotic properties (2, 3). In this regard, W. cibaria P71, which was previously isolated from common octopus (4), has been proposed as a suitable probiotic candidate for aquaculture, mostly due to its strong direct antimicrobial activity against turbot pathogens, resistance to bile, adhesion to mucus, and ability to inhibit the adhesion of turbot pathogens to mucus (2).

W. cibaria P71 was cultured on de Man-Rogosa-Sharpe (MRS) agar (1.5% [wt/vol]) plates (Oxoid, UK) at 30°C for 16 h. Subsequently, this strain was inoculated into sterile cryovials with cryogenic liquid and shipped to MicrobesNG (UK) for DNA sequencing. For DNA extraction, 25 μl of the suspension was lysed with 120 μl of Tris-EDTA (TE) buffer containing 0.1 mg/ml of lysozyme and 0.1 mg/ml of RNase A (ITW Reagents, Spain) and was incubated for 25 min at 37°C. Subsequently, 0.1 mg/ml of proteinase K (VWR Chemicals, USA) and SDS (0.5% [vol/vol]; Sigma-Aldrich, USA) were added and incubated for 5 min at 65°C. Then, DNA was purified using SPRI beads (Beckman, USA) and resuspended in elution buffer (EB) (Qiagen, Germany), and libraries were obtained using the Nextera XT library preparation kit (Illumina, USA) and the Microlab STAR automated system for liquid manipulation (Hamilton, USA). DNA libraries were quantified with a KAPA library quantification kit (Roche, Switzerland). Thereafter, whole-genome sequencing was performed using a NovaSeq 6000 system (Illumina) with the 250-bp paired-end sequencing protocol. Reads were analyzed with Trimmomatic v.0.33 (5), for adaptor removal. Reads with average Phred scores of ≥15 and lengths of >36 bp were *de novo* assembled using SPAdes v.3.11.0 (6). The quality of the assembled sequences was assessed using the QUAST v.5.0.2 tool (7). Coding DNA sequences (CDSs) were predicted and annotated using the NCBI Prokaryotic Genome Annotation Pipeline (PGAP) (8). The draft genome of W. cibaria P71 consists of 330 contigs (2,677,457 bp), with an *N*_50_ value of 351,779 and a G+C content of 44.4%. The total numbers of CDSs and RNAs were 2,622 and 118, respectively. Default settings were used for all software. During *in silico* analysis of the genome sequence, the BAGEL v.4.0 webserver (http://bagel4.molgenrug.nl) (9) predicted the absence of genes encoding bacteriocins, which agrees with our previous hypothesis that the antimicrobial activity exerted by W. cibaria P71 is due to organic acid production (10). A CRISPR array and two CRISPR-associated genes (*cas*) were identified (node 1) using the CRISPRCasFinder v.1.1.2 tool (https://crisprcas.i2bc.paris-saclay.fr/CrisprCasFinder/Index) (11). In BLASTn searches against the Virulence Factor Database v.5.0 (VFDB) (http://www.mgc.ac.cn/VFs/search_VFs.htm) (12) and analysis with the ResFinder v.4.1 tool (https://cge.cbs.dtu.dk/services/ResFinder) (13), genes encoding virulence factors and antibiotic resistance were not found.

The draft genome sequence of W. cibaria P71 presented here provides further data to support the safety and potential application of this lactic acid bacterial strain of aquatic origin as a suitable probiotic for aquaculture.

### Data availability.

This draft genome sequencing project has been deposited in DDBJ/ENA/GenBank under the accession number JAIBCW000000000. The version described in this paper is version JAIBCW010000000. The Sequence Read Archive (SRA) record is available under BioProject accession number PRJNA751720.
